# Determination and Validation of Zonisamide and its Four Related Substances by HPLC and UV-Spectrophotometry

**DOI:** 10.4103/0250-474X.70474

**Published:** 2010

**Authors:** Maryam Hosseini, E. Alipour, Arezou Farokhsir

**Affiliations:** Young researchers club, Islamic Azad University, North Tehran branch, Tehran, Iran; 1 Faculty of Chemistry, Islamic Azad University, North Tehran branch, Tehran, Iran; 2 Kimia Darou e Gharb Pharmaceutical Company, Shahid Salimi Industrial Site, Tabriz, Iran

**Keywords:** Dissolution, impurities, liquid chromatography, UV spectrophotometry, zonisamide

## Abstract

A selective and sensitive liquid chromatographic method has been developed for simultaneous determination of zonisamide and its four related substances in pharmaceutical dosage forms. The assay involved an isocratic elution in perfectsil Target C18 column using a mobile phase composition of disodium hydrogen phosphate buffer, acetonitrile and methanol (650:150:200 v/v, pH adjusted to 3±0.05) with flow rate 1.2 ml/min and analyte monitored at 240 nm. Also a simple and precise spectrophotometric method was developed for dissolution studies. These proposed methods are sensitive, accurate, reproducible and useful for the routine determination of zonisamide in pharmacy.

Zonisamide (1,2-benzisoxazole-3-methane sulfonamide,fig. 1) is used as an anticonvulsant in patients with epileptic disorders[1]. The exact mechanism of action is not known for zonisamide according to Leppik, while zonisamide may act as a carbonic anhydrase inhibitor like acetazolamide.

**Fig. 1:**
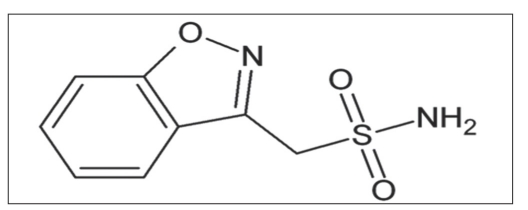
Structure of zonisamide

Several methods have been reported for the analysis of zonisamide using gas chromatography (GC)[1], micellar electrokinetic capillary chromatography[2,3] enzyme immunoassay[4], high performance liquid chromatography (HPLC) with UV detection using solid phase extraction[5] and HPLC method only for zonisamide by different methods[6–12]. No chromatographic method have been published till date for the quantitation of impurities, also no literature is reported for dissolution study by UV spectrophotometric method. In the present study, a rapid, specific, precise and validated HPLC (isocratic method) and UV spectrophotometric method for the quantitative estimation of zonisamide in pharmaceutical dosage forms is reported.

## MATERIALS AND METHODS

Zonisamide was obtained from Malladi Drugs and Pharmaceuticals, Chennai, India. Disodium hydrogen phosphates were obtained from Merck KGaA, Germany. HPLC grade acetonitrile and methanol were obtained from (Kaledon Labratories Ltd, Canada). All the other chemical reagents were analytical grade.

The HPLC system consisted of an Agilent Technology 1200 series, USA equipped with an Agilent Technologies G1311A Quat pump in a quaternary gradient mode and an Agilent Technologies1200 series VWD detector. Data aquisition was performed by the Chemstation software operated on a Pentium IV microprocessor. Analysis was Carried out at 240 nm, with a Perfectsil Target C18 reversed-phase column 250 4.6 mm i.d., 5 µm dimensions (MZ-Analysen Technik GmbH. Germany) at ambient temperature.

### Preparation of stock solutions, standard and QC samples:

Stock solution of zonisamide was prepared by dissolving accurately weighed 200 mg of the drug in 100 ml of mobile phase (final concentration 2 mg/ml). From this stock solution, standard 40 µg/ml was freshly prepared on the analysis day. Calibration standards were prepared at concentrations of 20 to 150 µg/ml from a standard solution of 200 µg/ml by appropriate dilution with mobile phase. Four quality control (QC) Samples at concentrations of (20, 50, 75 and 150%) representing the low, medium and high concentrations, respectively, of the linearity range were prepared from the standard solution.

For the estimation in dosage form, 20 tablets from each batch were randomly selected and powdered. Amount equivalent to 80 mg of zonisamide from powdered formulation was accurately weighed and taken in a volumetric flask, 40 ml of mobile phase was added, this mixture was subjected to vigorous shaking for 15 min for complete solution of the drug, then made up the volume to 50 ml, and then centrifuged at 4500 rpm for 30 min (Sigma 10l, Germany). Five milliliters of the clear supernatant was diluted 100 ml with mobile phase and 20 µl of this solution was injected for HPLC analysis.

### Linearity (calibration curve):

**Fig. 2:**
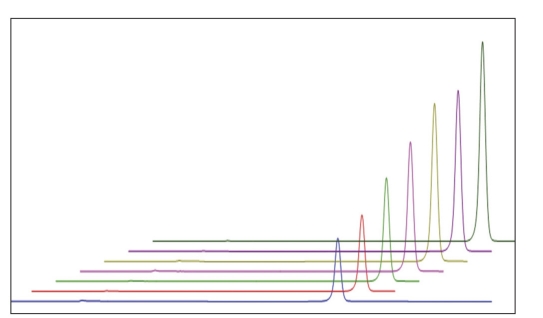
Chromatograms of zonisamide at different concentrations Chromatograms of zonisamide at different concentrations in the ascending order. Zonisamide elutes approximately at 8 min.

The calibration curves were constructed with eight concentrations including the LOQ ranging from 50 to 160 µg/ml of working concentration for zonisamaide and impurities [Fig. 2]. Each solutions was injected in three replicated and the linearity was evaluated by linear regression analysis, which was calculated by the least square regression method (Table 1).

**TABLE 1:** RESULTS OF REGRESSION ANALYSIS OF THE LINEARITY OF ZONISAMIDE BY HPLC

Parameter	Mean±SD
Slope	2.69976E-05
Intercept	0.00097019-
Correlation Coeffi cient (r ^2^)	0.00097019-

### Accuracy and precision (ruggedness):

Accuracy of the assay method was determined for both intra-day and inter-day variations using the triplicate analysis of the QC samples at three concentration levels (80, 100 and 120%) of working concentrations for zonisoamide. Precision of the assay was determined by repeatability (intra-day) and intermediate precision (inter-day). Repeatability refers to the use of the analytical procedure within a laboratory over short period of time that was evaluated by assaying the quality control samples during the same day and on different days using new solutions and different chromatographic systems. Intermediate precision was assessed by comparing the assay on different days for 3 days) (Table 2).

**TABLE 2:** COMPARISON OF INTRA-DAY AND INTERDAY PRECISION AND ACCURACY

Amount	HPLC method			UV spectrophotometric		
	Mean area	SD	RSD%	Mean area	SD	RSD%
Within day precision						
20	933.55	5.92	0.634	0.205	0.001	0.357
40	1576.12	1.09	0.069	0.229	0.001	0.069
60	2350.64	9.47	0.398	0.288	0.001	0.173
(Acceptance Criteria RSD<2)					
Between day precision						
20	923.843	5.591	0.601	0.204	0.001	0.283
40	1585.137	11.175	0.704	0.231	0.003	1.5
60	2355.138	12.788	0.542	0.286	0.004	1.415
(Acceptance Criteria RSD<3)					

Comparision of intra-day and inter-day precision and accuracy for the determination of zonisamide 100 mg by HPLC and UV-spectrophotometric methods

### Selectivity/Specificity

During Specificity study numbers of different solutions were prepared. Zonisamide, individual impurities, standard solution, sample solution and placebo solution were injected. The spectra and purity plots solutions were extracted through a diod array detector for each ingredient in the standard Furthermore, forced degradation studies were conducted in order to prove selectivity of the method.

### Robustness:

Several parameters of the method were purposely altered in order to determine the robustness of the method. The system suitability parameters as well as the recovery for the main ingredients in the sample solution were examined. The method parameters which altered were the columns temperature, the flow rate and the buffers pH [Table 3].

**TABLE 3:** ZONISAMIDE AND IMPURITIES IN SAMPLE SOLUTION AND SYSTEM SUITABILITY PARAMETERS THROUGH ROBUSTNESS STUDY

Method parameters	Impurity A	Impurity B	Impurity C	Impurity E	Zonisamide
working conditions	101.5	98.9	99.5	101.2	99.8
column temperature:30	100.2	97.4	98	99.5	100.02
column temperature:35	96.8	98.4	97.5	100.4	100.3
Flow rate:0.8 ml/min	95.8	97.8	101.6	96.8	100.23
Flow rate:1.4 ml/min	99.2	100.8	102.6	97.3	99.98
pH: 2.8			No Resolution		
pH: 3.2	100.5	103.8	97.7	101	98.87
Acceptance criteria	95%-105%	95%-105%	95%-105%	95%-105%	98%-102%

### System suitability:

Sample solution was injected three times in order to obtain the retention times of the components and all the important parameters of system suitability testing were calculated (RSD of area of zonisamide peak and h/v ratio of impurity B, where h is the peak height of impurity B and v is the distance between the top of the peak of impurity B and the lowest point of the valley defined between the peak due to impurity B and zonisamide).

### Recovery studies:

To check the accuracy of the developed methods and to study the interference of formulation additives, analytical recovery experiments were carried out by standard method. From the total amount of drug found, the percentage recovery was calculated. The results are 96.04-105.52±4.9, 0.28 (RSD).

### Detection and quantitation limits (Sensitivity):

Limits of detection (LOD) and quantitation (LOQ) were estimated from the signal-to-noise ratio. Detection limit was defined as the lowest concentration level resulting in a peak area of three times the baseline noise. The quantitation limit was defined as the lowest concentration level that provided a pick, with precision (% RSD) and accuracy (% bias) within ±10%.

### Stability of tablets and solutions:

The stability of the tablets was determined using the QC samples for short-term stability by keeping at room temperature for 36 h, and then analyzing. The long-term stability was determined by storing at 45򰤆 and 75% relative humidity (RH) for 90 days. The samples (n = 3) were taken out 30, 60, 90 days and evaluated for the drug content and physical parameters like color change, friability, hardness, and dissolution. Both standard and sample solutions were prepared and analyzed for recovery of zonisamide and four impurities at 0 h, 5 h, 12 h and 24 h at room temperature.

### Impurities:

**Fig. 3:**
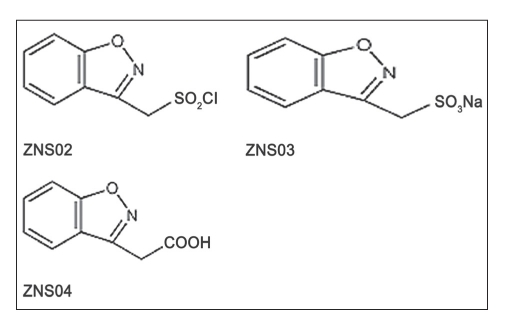
The structure of some ZNS related substances. ZNS03, sodium 1,2-benzisoxazole-3-methanesulfonate, is the structure of impurity E (RT is 4.6); ZNS04, 1,2-benzisoxazole-3-methanecarbonic acid, is the structure of impurity A (RT is 16.2); ZNS02, 1,2-benzisoxazole-3-methanesulfonylchloride is the structure of impurity B (RT is 11.32).

**Fig. 4:**
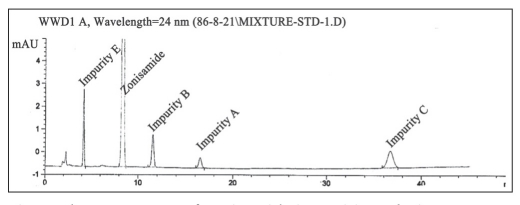
Chromatograms of zonisamide impurities solutions

Chemical structure of zonisamide impurities are shown in fig. 3. The stock solution of the impurities was prepared by dissolving accurately weighted 5 mg of their working standards in 20 ml of mobile phase (final concentration 0.25 mg/ml). From these stock solutions standard 0.01 mg/ml were prepared. The optimal composition of mobile phase was determined to be 25 mM disodium hydrogen phosphate buffer, acetonitrile and methanol (650:150:200 v/v, pH adjusted to 3±0.05). Flow rate was set at 1.2 ml/min. As can be seen in fig. 4, they have good resolutions and are well separated.

### Method validation:

Calibration curves were linear over the range of concentration used (50-120 µg/ml), which summarized in Table 4. Method precision, as indicated in [Table 5], was <2.5. The accuracy, as measured by the relative errors, ranged from 98.5 to 100.3% for all impurities. To check the accuracy of the developed methods and to study the interference of formulation additives, analytical recovery experiments were carried out by standard method. The results are reported in Table 5.

**TABLE 4:** LINEARITY RESULTS (N=3) FOR IMPURITIES OF ZONISAMIDE

Component	LOD (mg/ml)	LOQ (mg/ml)	R^2^	Regression equation
Impurity A	0.14	0.38	0.9999	y=25320208x-1291
Impurity B	0.12	0.35	0.9989	y=12899753x-1111
Impurity C	0.06	0.18	0.9995	y=18566832x-30001
Impurity E	0.15	0.37	0.9997	y=22032123x-1148

**TABLE 5:** ACCURACY, PRECISION AND RECOVERY RESULTS FOR IMPURITIS OF ZONISAMIDE

Parameters	Impurity A	Impurity B	Impurity C	Impurity E	Acceptance Criteria
Accuracy	99.80%	98.50%	100.03%	99.50%	95-105%
Precision	2.50%	0.85%	1.80%	0.56%	<5%
Recovery of standard	100.02	100.02	100.4	99.2	95-105%
Recovery of Sample	98.7	100.5	99.8	100.8	95-105%

**TABLE 6:** DISSOLUTION RESULTS BASED ON F1 AND F2 CALCULATIONS

Final Result	Standard
58.74: F2	≥50
F1: 4.51	≤15

### Dissolution studies:

**Fig. 5:**
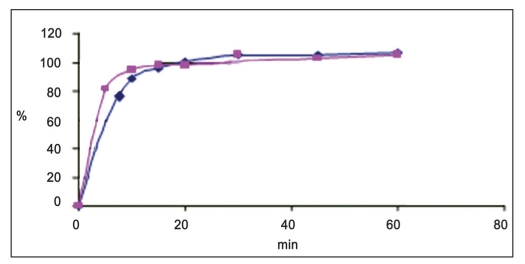
Comparison between sample and reference for dissolution study of zonisamide (
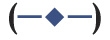
) Reference and (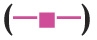) sample

Dissolution study was carried out using USP type 2 dissolution apparatus. The water bath, maintained at 37±0.5° and the paddle rotated at 50 rpm. At different time intervals, 15 ml sample was withdrawn, filtered the solution through Whatman filter paper No 42 then 5 ml of the resulting solution was transferred to 50 ml volumetric flask and volume was made up with 0.1 N HCl. The absorbance was measured at 240.2 nm by UV-Spectrophotometry. Release of zonisamide in reference and new formulation was same as each other and the values were calculated as follows (Fig. 5), (Table 6): $\mathrm{F1}\;=\;\sum \left(\left|\mathrm{Rt}-\mathrm{Tt}\right|\right)/\left(\sum \mathrm{Rt}\right)\times 100,$ $\mathrm{F2}\;=\;50\times \log\left\{\left[1+1/n\;\sum {\left(\mathrm{rt}-\mathrm{Tt}\right)}^{2}\right]-1/2\times 100\right\}$

### Validation for UV-Spectrophotometric method:

The calibration curves were constructed with eight concentrations from 50 to 160 µg/ml of working concentration for zonisamide. Each solution was measured in three replicates and the linearity was evaluated by linear regression analysis, which was calculated by the least square regression method. Linearity was obtained in this concentration range, with regression 0.9999, intercept -7.36724E-05 and slope 0.029701155, Accuracy of the assay method was determined for both intra-day and inter-day variations using the triplicated analysis of the QC samples and inter-day precision for the analyte was shown in Table 2. The intra and inter-day RSD were less than 2%. Also, during specificity study number of different solutions were prepared. Zonisamide, standard solution, sample solution and placebo solution were measured for specificity study.

## RESULTS AND DISCUSSION

Both, UV spectrophotometric and HPLC methods were found to be simple, accurate, economic and rapid for routine estimation of zonisamide in pharmaceutical dosage forms. For UV spectrophotometric method, in dissolution study, the value of standard deviation and RSD in recovery (0.285< 2%); shows the high precision of the method.

For the assay method, HPLC conditions were optimized to obtain, an adequate separation of eluted compounds. Mobile phase and flow rate selection was based on peak parameters (height, tailing, theoretical plates, capacity factor), and run time. In separation of impurities Compounds with similar structure elute close to each other. Parameters that effect retention and separation are percentage of organic solvent and type of organic solvent, because they defined the polarity of the mobile phase and kind of interaction between molecules and stationary phase. Different percentages of mobile phases were tried in order to achieve the best separation. To ascertain its effectiveness, system suitability tests were carried out on freshly prepared stock solutions. Sample- to sample precision and accuracy were evaluated using, three samples of three different concentrations, which were prepared and analyzed on same day. Day-to-day variability was assessed using three concentrations analyzed on three different days, over a period of two weeks. Thus, it was concluded that there was no significant difference on the assay, which was tested on an intra-day and inter-day basis. The proposed methods are accurate, simple, rapid and selective for determination of zonisamide and especially for its impurities with the same conditions, in pharmaceutical dosage forms. Hence, it can be conveniently adopted for the routine quality control analysis.

The rapidity of the assay allows analysis of a large number of samples with less mobile phase, which proves to cost-effective. Furthermore, spectrophotometric method for dissolution is so time consuming. Compared with other published methods, The HPLC assay in this article is faster and simpler and allows determining very small amounts of zoniszamide.
